# Label-free quantitative proteome data associated with MSP1 and flg22 induced signaling in rice leaves

**DOI:** 10.1016/j.dib.2018.07.063

**Published:** 2018-07-31

**Authors:** Qingfeng Meng, Ravi Gupta, Cheol Woo Min, Jongyun Kim, Katharina Kramer, Yiming Wang, Sang-Ryeol Park, Iris Finkemeier, Sun Tae Kim

**Affiliations:** a Department of Plant Bioscience, Life and Energy Convergence Research Institute, Pusan National University, Miryang, 627-706, South Korea; b Division of Biotechnology, Korea University, Seoul, 02841, South Korea; c Plant Proteomics Group, Max Planck Institute for Plant Breeding Research; d Department of Plant-Microbe Interactions, Max Planck Institute for Plant Breeding Research, Carl-von-Linné Weg 10, 50829 Cologne, Germany; e Gene Engineering Division, National Institute of Agricultural Sciences, Rural Development Administration, Jeonju 54874, South Korea; f Institute of Plant Biology and Biotechnology, University of Muenster, Schlossplatz 7, 48149 Muenster, Germany

## Abstract

The data set reported here is associated with the article “A proteomic insight into the MSP1 and flg22 induced signaling in *Oryza sativa* leaves”. MSP1, a cerato-platanin protein, induces cell death and triggers PAMP (pathogen-associated molecular pattern)-induced immunity PTI in rice [1]. To understand the MSP1 induced PTI signaling in rice, we performed a high-throughput proteome analysis combined with PLS-DA (partial least squares discriminant analysis) and qPCR.

**Specifications Table**

**Table:** 

Subject area	Biology
More specific subject area	Plant Science, Proteomics, Plant pathogen interaction, PAMP induced immunity
Type of data	Tables and figures
How data was acquired	EASY-nLC 1000 (Thermo Fisher, USA) coupled with QExactive Plus mass spectrometer (Thermo Fisher, USA), Rotor-Gene Q instrument (QIAGEN, Hilden, Germany)
Data format	Raw, analyzed
Experimental factors	PAMP induced immunity
Experimental features	Shotgun proteome analysis of rice leaves in response to MSP1 and flg22 treatments
Data source location	Plant Proteomics facility of the Max Planck Institute for Plant Breeding Research Cologne, Germany
Data accessibility	Data are available within this article

**Value of the data**

● The data set reported here was obtained from the proteome analysis of rice leaves in response to control (ddH_2_O), MSP1 and flg22 treatment, which contributes to our understanding of MSP1 and flg22 triggered PTI signaling in rice.
● MaxQuant label-free proteome analysis led to the identification of 4167 protein groups with 433 differential proteins in response to MSP1 and/or flg22 treatment, which would be shared with the other research groups for further study and could be used as a resource to understand the *Magnaporthe oryzae* induced signaling in rice
● qPCR of 20 signaling proteins were carried out, providing the changes at the transcript level after MSP1 and flg22 treatment.

## 1 Data

**Fig. 1:**
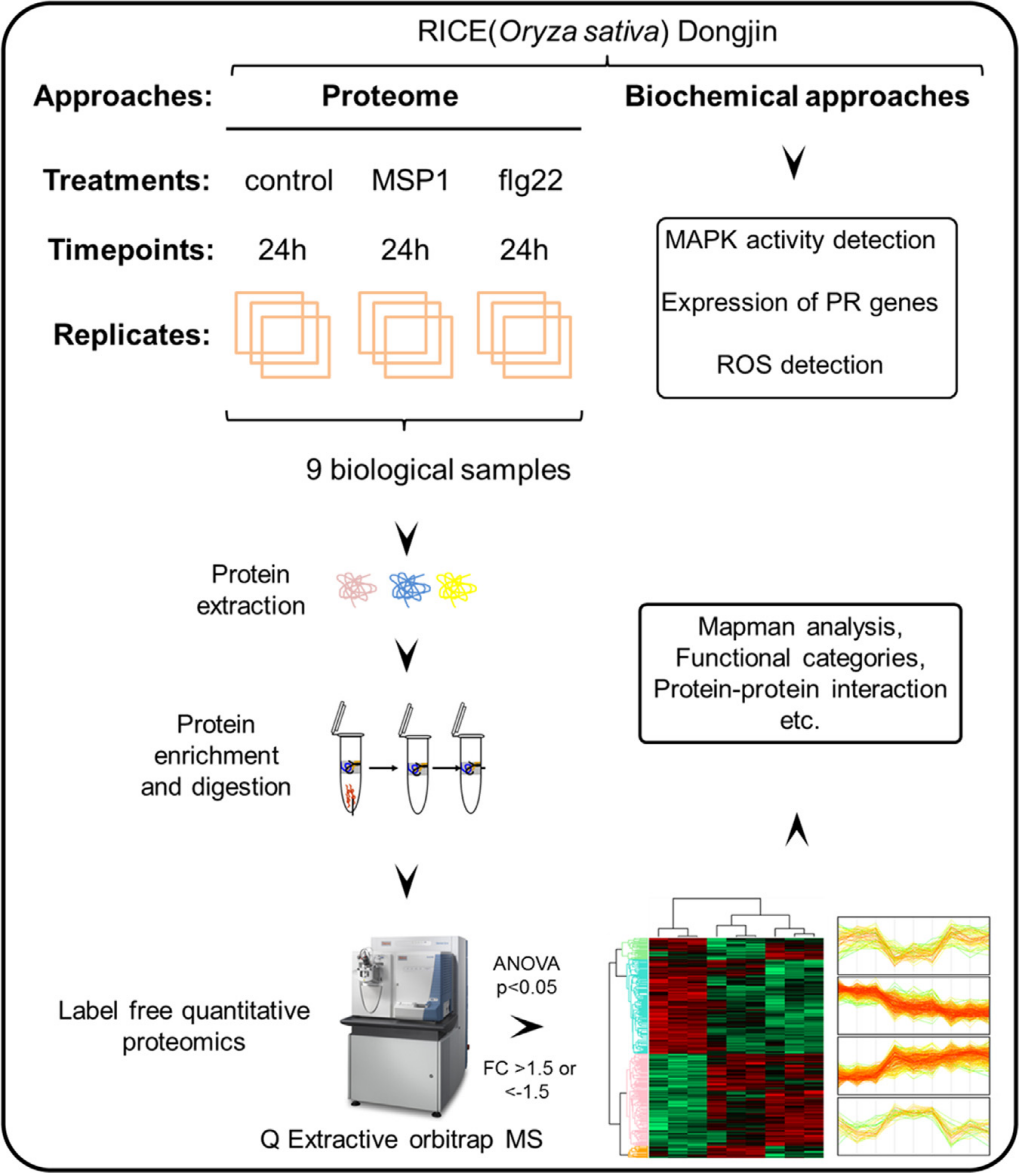
Experimental procedure of sample preparation and data analysis.

**Fig. 2:**
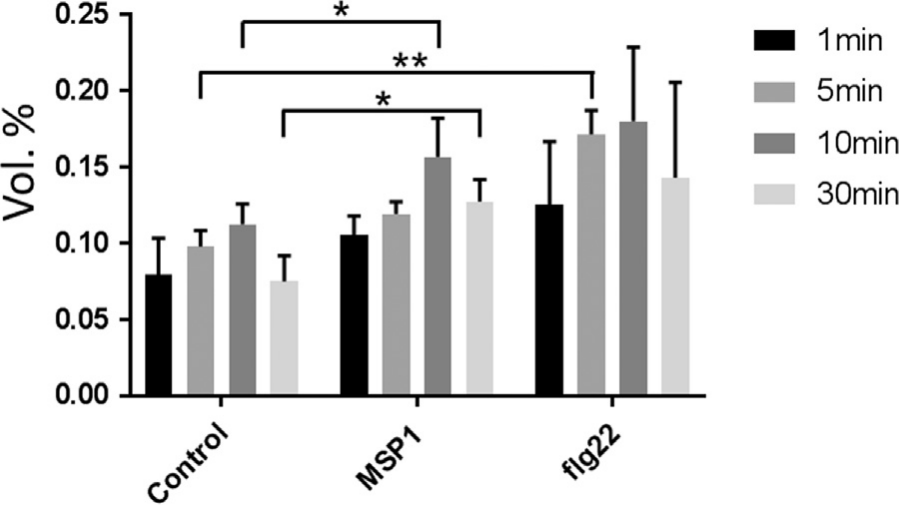
Quantification of MAPKs assay with three independent replicates. * shows significant difference, *, *p* <0.05; **, *p* <0.01.

**Fig. 3:**
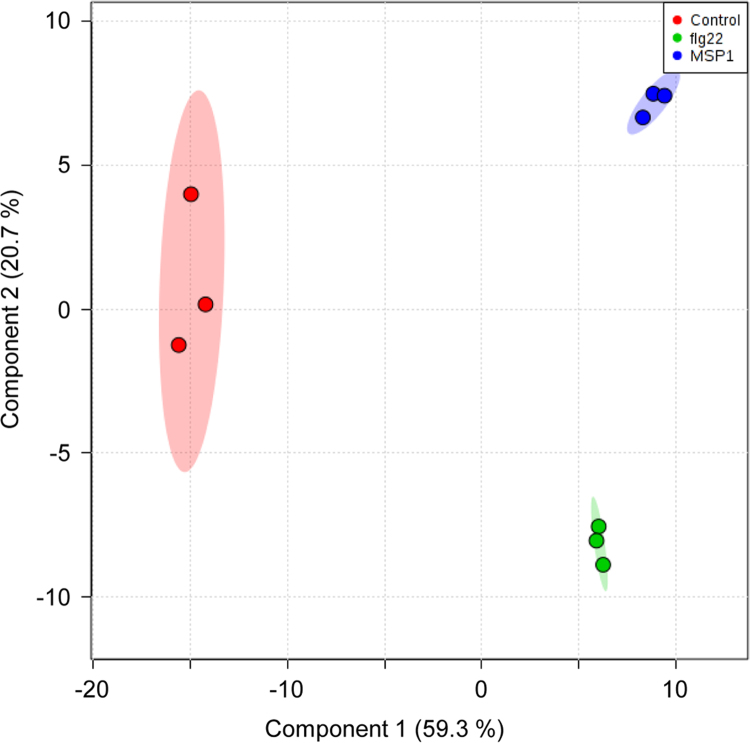
PLS-DA analysis of DMPs in MSP1 and flg22 treated sample.

**Fig. 4:**
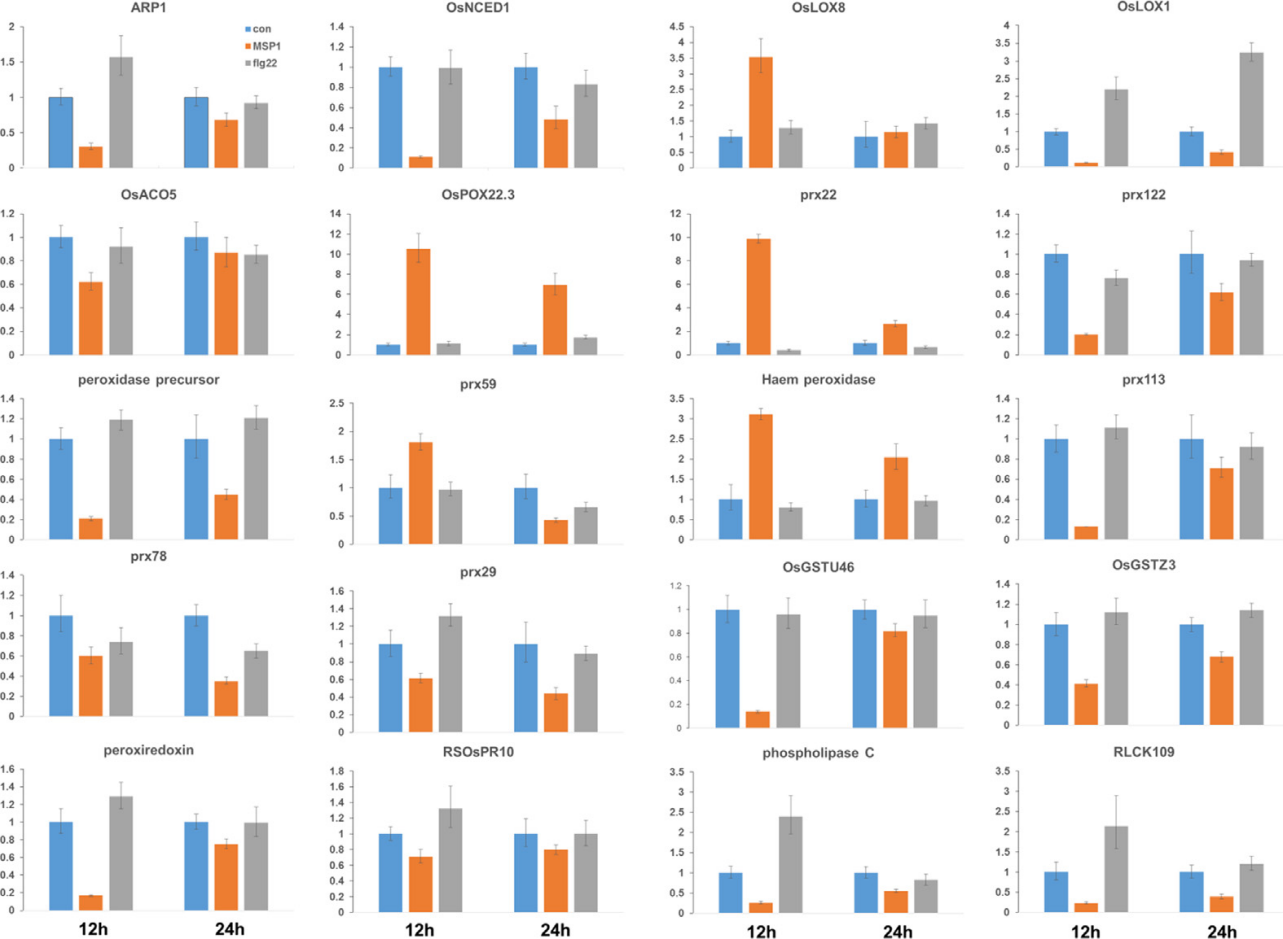
Expression profile of 20 signaling proteins achieved by qPCR. Relative level of transcript was normalized to control (con) samples. Error bars represent the standard deviation obtained from three replicates.

Figures reported here depict experimental workflow (Fig. 1), MAPK (mitogen-activated protein kinase) assay (Fig. 2), PLS-DA analysis (Fig. 3) and qPCR (Fig. 4) of the rice leaves treated with MSP1 and flg22. Supplementary Tables show the list of primers used in qPCR (Supplementary Table 1), proteins differentially modulated by MSP1 and flg22 (Supplementary Table 2 and 3), functional annotation of the differential proteins (Supplementary Table 4), biotic stress related proteins (Supplementary Table 5), protein-protein interaction network (Supplementary Table 6), and qPCR results (Supplementary Table 7). PLS-DA scores plot showed that control and MSP1/flg22 samples were clearly separated to each other in component 1 showing 59.3% of total variance. While separation of MSP1 and flg22 samples observed in component 2 shows only 20.7% of total variance, suggesting lesser differences in the protein profiles between these two samples. Detailed description of the data and methods is reported previously [1].

## 2 Experimental design, materials and methods

### 2.1 Plant material and sample preparation

Rice seeds (*Oryza sativa* L. Dongjin) were planted to sterilized soil in a growth chamber (70% humidity, 25 °C; a light and dark cycle of 14 and 10 h, respectively) for 4 weeks as described previously [1]. Then, 4-week-old rice plants were sprayed with ddH_2_O (control), 1 μM purified recombinant MSP1-His protein, or 1 μM synthetic bacterial flagellin peptide flg22 (LugenSci, South Korea). Each treatment was performed in three replicates (6 × 3 = 18 rice plants/treatment) and the treated secondary leaves were harvested at 24 h post-treatment (hpt), pooled together, powdered using liquid nitrogen and stored at − 70 °C until further analysis.

### 2.2 Protein extraction

For proteome analysis, total proteins were extracted from control, MSP1, and flg22 treated leaves and protamine sulfate (PS) precipitation method was used for detection of low-abundance proteins (LAPs) as described previously [1].

### 2.3 Sample preparation and LC–MS/MS for proteomic analysis

Sample preparation and trypsin digestion were carried out as described in reference [1]. Peptides were separated with an EASY-nLC 1000 (Thermo Fisher) on a 16 cm C18 column (3 µm bead size) and analyzed with a QExactive Plus mass spectrometer (Thermo Fisher) using Top15 method as described previously [1]. The acquired MS data were analyzed with MaxQuant (ver. 1.5.3.12) [2] and perseus software (ver. 1.5.8.5) [3] as described previously [1].

### 2.4 Bioinformatics analysis

Proteins were classified into MapMan BINs and their annotated functions were visualized using the MapMan program by searching against *O. sativa* Osa_MSU_v7 mapping [4]. Interactome analysis was performed using Cytoscape [5] combined with STRING application as described previously [5]. PLS-DA analysis was carried out by online tool MetaboAnalyst as described previously [1].

### 2.5 RNA isolation and qPCR analysis

Total RNA was isolated and reverse transcripted into cDNA as described previously [1]. qPCR was performed using the Rotor-Gene Q instrument (QIAGEN, Hilden, Germany) with a QuantiNova SYBR Green PCR kit (QIAGEN, Hilden, Germany) as described previously [1].

## Appendix A Supplementary material

Table 1. Primer sequences used for qPCR analyses.

.

Table 2. Differentially modulated proteins by MSP1 or flg22 treatment.

.

Table 3. Overall analysis of differentially modulated proteins.

.

Table 4. Functional overview of differentially regulated proteins.

.

Table 5. Biotic stress–related proteins altered by treatment.

.

Table 6. Network of differentially modulated proteins by MSP1 treatment.

.

Table 7. Gene expression levels detected by proteomics approach and qPCR

## Footnotes

Appendix A Supplementary data associated with this article can be found in the online version at https://doi.org/10.1016/j.dib.2018.07.063.
