# Supplementary material for: Label-free quantitative proteome data associated with MSP1 and flg22 induced signaling in rice leaves
Source: Data Brief. 2018 Jul 31;20:204–9. doi: 10.1016/j.dib.2018.07.063 (PMC6097273; doi:10.1016/j.dib.2018.07.063)
Supplement: Supplementary file 1 — Transparency document [file mmc1.docx]

**Conflict of Interest**

The authors have declared that no competing interests exist.
